# Huntingtin and its allies at the cortico-striatal synapse

**DOI:** 10.1038/s41419-026-08584-6

**Published:** 2026-03-27

**Authors:** Chiara Zuccato, Andrea Scolz, Raffaele Iennaco

**Affiliations:** 1 Department of Biosciences, University of Milan, Milan, Italy; 2 Istituto Nazionale di Genetica Molecolare “Romeo ed Enrica Invernizzi”, Milan, Italy

**Keywords:** Protein-protein interaction networks, Synaptic plasticity, Huntington's disease, Synaptic transmission, Mechanisms of disease

## Abstract

Huntington’s Disease (HD) is characterized by progressive motor and cognitive decline, largely driven by cortico-striatal synaptic dysfunction. Central to these processes is huntingtin (HTT) protein, which is abundantly present at the synapse. HTT regulates the synaptic vesicle cycle at presynaptic terminals and serves as a scaffold at the postsynaptic density where it modulates receptor dynamics. An expanding network of HTT-interacting proteins (HIPs), crucial for maintaining synaptic structure and function, underscores the role of HTT as a core component of synaptic integrity. This review examines the 30-year research journey that has unveiled HTT pre- and postsynaptic partners, with focus on experimentally validated interactors and their involvement in HD cortico-striatal synaptic dysfunction.

## Facts

- Huntingtin (HTT) interacts with over 3400 proteins, highlighting its central role in a wide array of cellular processes.
- A significant proportion of the Huntingtin interacting proteins (HIPs) are synaptic proteins.
- Many validated HIPs are essential for axonal transport and the synaptic vesicle cycle, emphasizing the importance of HTT in the presynaptic machinery.
- Presynaptic dysfunction at the cortico-striatal synapse drives neuronal degeneration, suggesting that presynaptic HIPs are promising therapeutic targets in Huntington’s Disease.

## The cortico-striatal synapse in Huntington’s disease: a critical hub of pathology

Synapses are fundamental units of neuronal communication, orchestrating the precise transmission of information across neural circuitries. The integrity of synaptic function is essential for normal brain activity, and even subtle perturbations can have profound consequences, often manifesting as neurological or psychiatric disorders. An increasing body of evidence implicates synaptic dysfunction as a central contributor to the pathogenesis of Huntington’s Disease (HD), a devastating autosomal dominant neurodegenerative disorder caused by the expansion of a CAG trinucleotide repeat beyond 36 units within the *huntingtin* (*HTT*) gene [1]. This pathogenic expansion is prone to progressive somatic instability, resulting in striking mosaicism across brain neurons [2]. In particular, the accumulation of expanded CAG repeats, especially those exceeding a critical threshold of ~150 units, disrupts neuronal homeostasis, leading to synaptic failure, neuronal dysfunction, and selective neurodegeneration, most prominently within the cortex and striatum [2–5]. These two regions are interconnected *via* the cortico-striatal pathway, a glutamatergic circuitry linking layer V cortical pyramidal neurons (CPNs) to striatal GABAergic medium spiny neurons (MSNs) [6]. This pathway serves as a central hub where cognition, emotion, and movement converge, and is essential for motor planning, execution, and goal-directed behavior [6]. Multiple lines of evidence show that the cortico-striatal synapse is one of the earliest and most selectively affected sites in HD pathophysiology [6]. Post-mortem analyses of HD brains revealed that degeneration of this synapse occurs from the initial stages of the disease [7]. Longitudinal neuroimaging studies in premanifest and early symptomatic HD patients highlighted a strong correlation between cortico-striatal disconnection and clinical progression [8]. This synaptic breakdown is accompanied and exacerbated by non-neuronal players. Dysfunctional astrocytes and overactive microglia contribute to synaptic loss, particularly within the cortico-striatal circuitry, further highlighting the vulnerability of this synapse in HD [7, 9]. In vivo evidence obtained from mouse models also supports the involvement of the cortico-striatal circuitry in HD pathogenesis. Conditional deletion of mutant HTT from MSNs and CPNs—while preserving its expression elsewhere in the brain—ameliorates behavioral deficits and brain atrophy in a mouse model of HD [10]. Notably, subsequent findings demonstrated that expression of mutant HTT in CPNs is a key component of cortico-striatal dysfunction and behavioral phenotypes in HD mice [11]. More recently, the reconstruction of the cortico-striatal circuitry in microfluidic chambers has confirmed that the presence of mutant HTT in cortical neurons is necessary and sufficient to alter the functionality of the circuitry and to induce striatal atrophy [12, 13]. These findings challenge the classical paradigm that positions the striatum as the primary site of pathology in HD, instead implicating presynaptic cortical dysfunction as a key driver of the degenerative cascade.

A critical question thus arises: how does mutant HTT perturb the molecular dialogue between cortical and striatal neurons? Given the synaptic localization of HTT, numerous studies have endeavored to elucidate the molecular mechanisms by which its mutant form disrupts the pre- and postsynaptic machineries. At the core of this research lies the systematic mapping of the HTT protein interaction network. Thus far, over 3400 putative huntingtin-interacting proteins (HIPs) have been identified (see Table 1). Notably, meta-analyses of the HTT interactome datasets revealed that a substantial proportion of HTT partners are involved in the organization and maintenance of synaptic architecture [14–17]. Collectively, these observations support the view of HTT as a synapse-organizing protein.

**Table 1:** Navigating HIPs: the HTT protein-protein interaction databases.

Web site	URL	Key features	
HDinHD	https://www.hdinhd.org/	• HIPs from >230 publications • Experimental metadata for each HIP • Possibility to filter by HTT variant, model, detection method • Integrated transcriptomic & proteomic datasets	[195]
HTT-OMNI	http://htt-omni.princeton.edu:5006/	• ~3,400 HTT interactors from HINT (https://hint.yulab.org/) • Systems-level omics analysis in HD models • Links to known HD genetic modifiers • Tools for visualizing new HIP datasets	[17]

In this review, we delve into the pre- and postsynaptic subsets of validated HIPs, examining how their interactions with HTT – under both physiological and pathological conditions – inform our understanding of the mechanisms by which mutant HTT compromises cortico-striatal communication. By delineating this molecular network, we aim to identify direct and indirect HTT interactors that contribute to synaptic vulnerability in HD and represent promising targets for therapeutic intervention.

## Mapping presynaptic hips

In this section, we examine how HIPs regulate presynaptic function, with a particular focus on their role in axonal transport and in coordinating the synaptic vesicle (SV) cycle.

### Driving (car)go along axons

Axonal transport is an ATP-dependent and bidirectional mechanism that enables the movement of diverse cargoes along microtubules between the soma and distal synaptic terminals [18]. This process is critical for synaptic integrity and neuronal viability as it ensures the targeted delivery and clearance of organelles, protein complexes, and signalling molecules required for neurotransmission [18]. The kinesin superfamily of motor proteins is responsible for anterograde transport of synaptic vesicle precursors (SVPs) and organelles from the cell body towards the growth cones and synapses [18]. Retrograde transport towards the soma, on the other hand, is mediated by the dynein-dynactin complex and serves as a critical feedback mechanism that triggers changes in gene expression, and enables the clearance of damaged synaptic components [18].

**Fig. 1:**
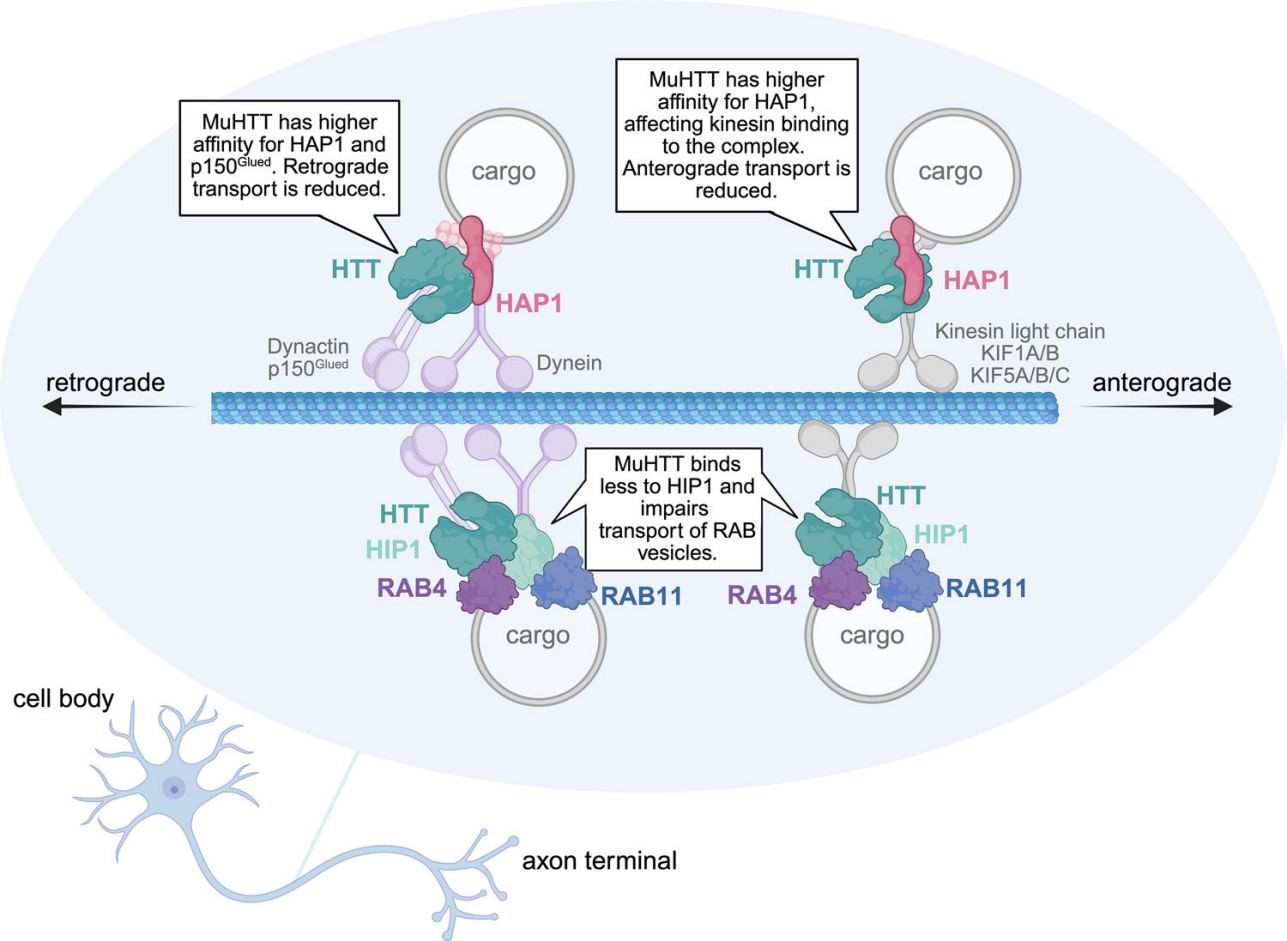
HTT partners in the axonal transport machinery. HTT regulates axonal transport through interactions with two adaptor proteins of the transport machinery: HAP1 and HIP1. HTT binds to kinesin light chain and kinesin heavy chain isoforms KIF1A/B and KIF5A/B/C, which mediate anterograde transport. It binds also to p150^Glued^, a subunit of the dynactin complex, and to the dynein intermediate chain, which mediate retrograde transport. The HTT-HIP1 complex engages Rab GTPases, master regulators of intracellular vesicular trafficking. Mutant HTT (muHTT) disrupts the assembly of HTT–HAP1 and HTT–HIP1 motor complexes, thereby impairing axonal trafficking. *Created in* https://BioRender.com/r55y183.

HTT occupies a central position in the axonal transport machinery through its coordinated interactions with cytoskeletal proteins and components of the motor complex. By associating with microtubules and binding directly to F-actin, HTT organizes the cytoskeletal architecture required for efficient cargo movement along axons [19]. A substantial body of evidence indicates that HTT exerts its function in axonal trafficking [20–23] through two key HIPs: huntingtin-associated protein 1 (HAP1) [24, 25] and huntingtin-interacting protein 1 (HIP1) [26, 27] (Fig. 1), both of which bind core components of the transport machinery. Moreover, the role of HTT in trafficking is reinforced by its ability to recruit the glycolytic enzyme glyceraldehyde-3-phosphate dehydrogenase (GAPDH) to vesicular membranes, thereby enabling localized ATP production that sustains continuous vesicle motility along microtubules [28].

HAP1, the first interactor of HTT identified *via* yeast two-hybrid screening in 1995, is ubiquitously expressed in the central nervous system (CNS) and localizes to the cytoplasm, axons, and dendrites, where it binds to microtubules [24, 25]. Studies in the mouse brain have demonstrated that HTT forms a multiprotein complex with HAP1, which in turn recruits components of the kinesin-1 motor complex, including kinesin light chain, the heavy chain isoforms KIF5A, KIF5B, and KIF5C, and also p150^Glued^, a subunit of the dynactin complex [23, 29–31]. HTT interaction with KIF5C and KIF2A has been demonstrated also in an immunoprecipitation-mass spectrometry (IP-MS) study performed on the mouse cortex, further attesting to the central role of HTT in axonal transport [15]. Additionally, HTT has been shown to directly interact with the dynein intermediate chain, as evidenced by yeast two-hybrid and GST pull-down assays, which mapped the interaction domains to amino acids 1–283 of dynein and 600–698 of HTT [23]. GST-HAP1 pull-down and co-immunoprecipitation experiments in HD cell lines revealed that mutant HTT has an abnormally high affinity for HAP1 and p150^Glued^ [22, 24]. Such aberrant interactions disrupt the integrity of the axonal transport machinery, causing cargo to detach from microtubules and ultimately impairing bidirectional transport.

First identified in 1997 as a HTT interactor involved in clathrin-mediated endocytosis in the cerebral cortex [26, 27], HIP1 links HTT to vesicles containing Rab-GTPases, a family of small GTPases that orchestrate intracellular trafficking with high specificity and directionality [32, 33]. Co-immunoprecipitation studies in mouse brains and induced pluripotent stem cells (iPSCs)-derived human neurons confirmed the presence of HTT-Rab4 and HTT-Rab11 complexes, which rely on kinesin-1 and dynein for axonal transport, with HIP1 acting as an adaptor [32]. In vivo studies in *Drosophila* larval axons showed that HTT co-localizes and co-migrates with Rab2, Rab3, Rab7, and Rab19 vesicles [34]. Subsequent HTT interactome studies performed on the cortex, striatum, and cerebellum from wild-type and heterozygous mutant HTT knock-in mice revealed a broader set of Rab GTPases interacting with HTT, highlighting the complexity of its role in vesicular trafficking and the need for further investigation [14]. Together, these findings support a model in which HTT facilitates axonal transport by linking Rab GTPases to motor proteins through HIP1. Notably, yeast two-hybrid studies have shown that mutant HTT exhibits reduced binding affinity for HIP1 [26], which can impair Rab-mediated vesicle trafficking in HD.

HTT orchestrates trafficking of a diverse array of cargoes, including synaptic precursors [35] and neurotransmitter receptors [31], brain-derived neurotrophic factor (BDNF)-containing vesicles [22], mitochondria [36–40], autophagosomes [41], as well as endosomes and lysosomes [42, 43]. Among the repertoire of HTT-associated cargoes, mitochondria and BDNF-containing vesicles have been among the most intensively studied, due to their essential contributions to neuronal function and synaptic integrity.

#### Tracking mitochondria

Mitochondria are actively trafficked along axons to presynaptic terminals, where they sustain neurotransmission by supplying locally generated ATP and buffering intracellular Ca²⁺ for efficient SVs cycling and exocytosis [44, 45]. Although the role of wild-type HTT in mitochondrial transport has been debated [22, 36], multiple studies have demonstrated that mutant HTT disrupts anterograde mitochondrial trafficking in HD cortical neurons [36–40]. In vitro reconstructions of the HD cortico-striatal circuitry have revealed that mitochondrial trafficking deficits emerge as a late-stage pathological event, particularly affecting the transport of small mitochondria produced by fission along cortical afferents [13]. Studies in mouse brains and human tissues showed that HTT binds to the outer mitochondrial membrane dynamin-related protein 1 (Drp1), a key effector of fission [39, 46]. By contrast, mutant HTT exhibits aberrantly high affinity for Drp1, leading to its hyperactivation and resulting in excessive mitochondrial fragmentation [39, 46]. In HD, fragmented mitochondria show reduced axonal motility, possibly due to disrupted interactions with Miro Rho-GTPases that regulate mitochondrial transport along microtubules [47, 48]. Mutant HTT also binds to the translocase of the inner mitochondrial membrane complex TIM23, a machinery responsible for importing nuclear-encoded proteins into mitochondria [49]. GST pull-down and co-immunoprecipitation assays, performed in vitro and in the brains of HD transgenic mice, demonstrated that mutant HTT displays higher affinity than wild-type HTT for key TIM23 subunits, including Tim50, Tim23, and Tim17 [49, 50]. This pathological interaction impairs mitochondrial protein import, resulting in widespread alterations of the mitochondrial proteome [49, 50]. Given that approximately 99% of mitochondrial proteins are encoded by the nuclear genome, even subtle perturbations in TIM23-dependent import can compromise mitochondrial integrity. Altogether, these findings indicate that, in HD, mitochondria accumulate in the soma, while synaptic terminals remain sparsely populated by a limited pool of small and bioenergetically compromised organelles [44, 45, 51].

#### Leading the BDNF journey

Deficits in BDNF trafficking are particularly detrimental within the cortico-striatal pathway in HD. Notably, the striatum does not produce significant levels of endogenous BDNF, and therefore relies heavily on anterograde transport of BDNF synthesized in cortical neurons [52–54]. Once delivered to the striatum *via* cortico-striatal afferents, BDNF supports the survival, development, and synaptic plasticity of MSNs through activation of the Tropomyosin receptor kinase B (TrkB) [55]. Reduced BDNF trafficking, compounded by transcriptional downregulation of the BDNF gene, results in insufficient replenishment of the presynaptic cortical BDNF pool [12, 13, 22, 56–59]. This dual mechanism deprives striatal MSNs of critical trophic support, ultimately driving disconnection and degeneration of the cortico-striatal circuitry [22, 57–59]. Consequently, therapeutic strategies aimed at restoring cortical BDNF production and transport have emerged as promising approaches for HD treatment [58, 59]. Advancements in microfluidic platforms have enabled precise in vitro modelling of the cortico-striatal circuitry and high-resolution live imaging of BDNF dynamics in cortical axons, revealing that mutant HTT reduces the velocity and the number of BDNF-containing vesicles in both anterograde and retrograde directions [12, 13, 60]. Post-translational modifications (PTMs) of HTT fine-tune its interaction with motor proteins, ultimately dictating the direction and speed of BDNF vesicle transport. Methylation at arginine 118 by Protein Arginine Methyltransferase 6 (PRMT6) promotes HTT recruitment to the transport machinery, and anterograde BDNF trafficking in cortical afferents [61]. Phosphorylation at serine 421 of HTT also regulates the directionality of BDNF transport. When serine 421 is phosphorylated, HTT recruits kinesin-1 to the dynactin complex on vesicles and promotes BDNF anterograde transport [62]. Conversely, when serine 421 is unphosphorylated – as occurs in HD [63] – kinesin-1 detaches and vesicles are more likely to undergo retrograde transport [62], reducing bioavailability of BDNF at the presynaptic terminal. Furthermore, cyclin-dependent kinase 5 (Cdk5) phosphorylates HTT at serine 1181 and serine 1201 to detach vesicles and motor complexes from microtubules, thereby reducing BDNF transport in both anterograde and retrograde directions [64]. These findings not only reinforce the connection between HTT and the BDNF transport machinery, but also underscore the potential role of PTMs as key modulators of HTT interactions with binding partners. This domain represents a largely uncharted area of research that warrants deeper investigation.

### HIPs tie HTT to the SV cycle

Presynaptic compartments are distinguished by the presence of hundreds of neurotransmitter-filled synaptic vesicles (SVs) and active zones (AZs), which are specialized regions of the presynaptic plasma membrane where SVs are recruited for release [65]. Neurotransmitter secretion is a highly coordinated multi-step process that involves SVs recruitment to the synapse, exocytosis, and subsequent recycling, all of which are intimately connected to form the SV cycle [65]. Each stage of this cycle is tightly regulated by dynamic protein complexes [65]. HTT localizes to SVs [66, 67], and contributes to the SV cycle by binding to proteins involved in the molecular machineries that guide SVs recruitment to the AZ, exocytosis, and recycling.

#### Governing SVs recruitment at the AZ

**Fig. 2:**
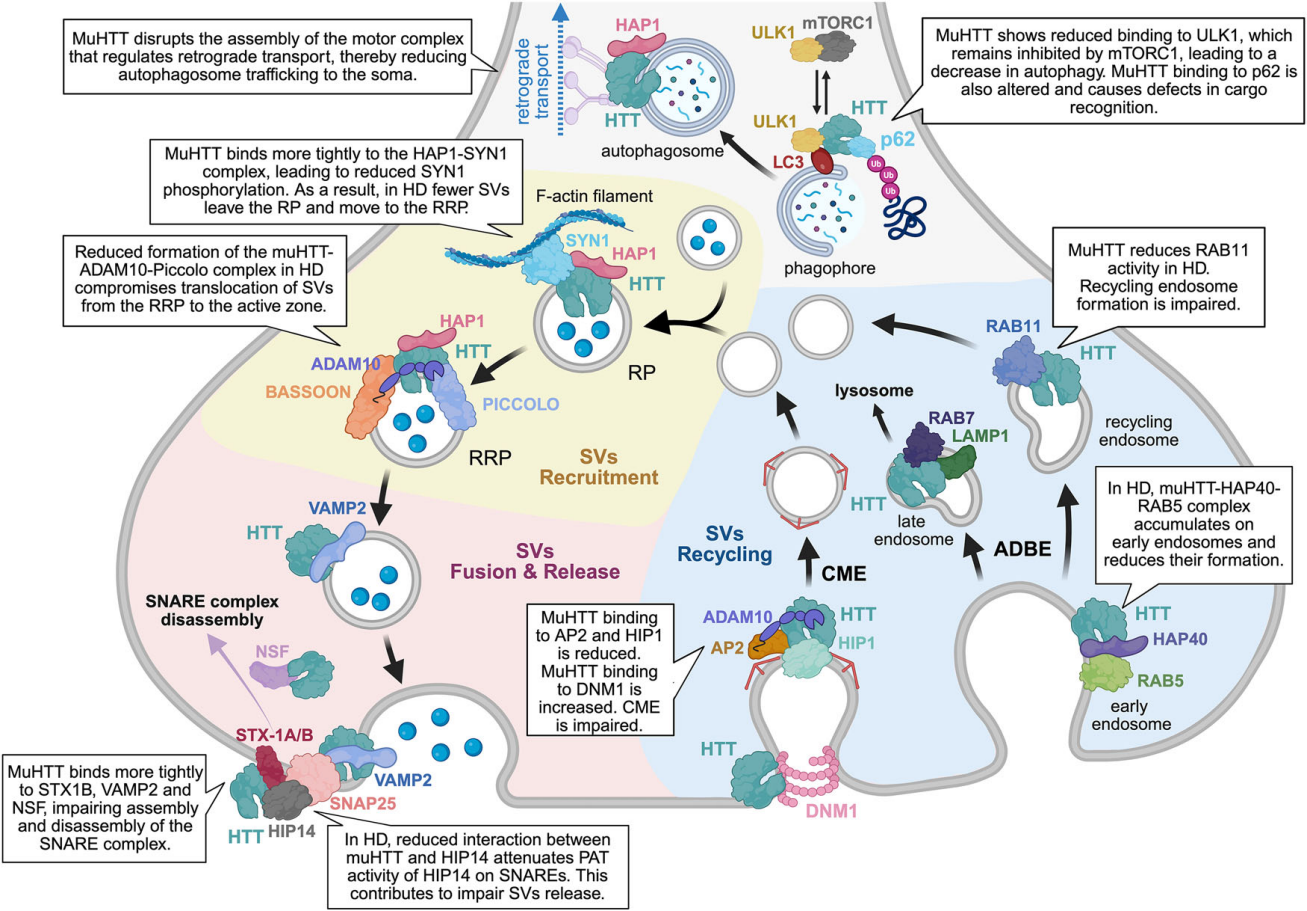
HTT interacting proteins in synaptic vesicle turnover and autophagy. HTT localizes to synaptic vesicles (SVs) and participates in protein complexes involved in SVs recruitment (yellow background), fusion and release (pink background), recycling (light blue background), and autophagy (grey background). Expansion of the polyQ tract in HTT disrupts the assembly of these complexes, impairing the SV cycle and autophagy. Abbreviations: RP (reserve pool), RRP (ready releasable pool), CME (clathrin-mediated endocytosis), ADBE (activity-dependent bulk endocytosis). *Created in* https://BioRender.com/r55y183.

The presynaptic bouton is densely packed with ∼200-300 vesicles, only a small fraction of which are mobilized for immediate release [68]. These vesicles, known as the ready releasable pool (RRP), are docked at the AZ and poised for immediate release upon the arrival of action potentials [68]. To sustain continuous neurotransmitter release, the RRP must be constantly replenished by newly recruited SVs from a larger reserve pool (RP) located distally to the AZ [68]. As highlighted by recent studies, HTT interacts with a variety of presynaptic proteins to facilitate the recruitment of SVs in the RRP (Fig. 2).

Synapsins represent a family of highly conserved phosphoproteins that interact with SVs in the presynaptic bouton [69, 70]. In particular, the neuron-specific phosphoprotein synapsin 1 (SYN1), by binding to SVs and F-actin filaments, is essential to maintain SVs in the RP [69, 70]. Upon phosphorylation by the mitogen-activated protein kinase (MAPK) pathway, SYN1 dissociates from the vesicles that translocate from the RP to the RRP for subsequent release [69, 70]. Both HTT and its adaptor protein HAP1 interact with SYN1 to regulate this critical presynaptic step (Fig. 2). Co-immunoprecipitation and interactomic analyses have shown that wild-type HTT forms a complex with SYN1 in the mouse cortex, with this interaction being enhanced in the context of HD [16, 71]. Mutant HTT exhibits increased affinity for the proline-rich C-terminal domain of SYN1, which is crucial for SVs trafficking, thereby reducing SYN1 phosphorylation and SVs mobilization towards the RRP [71]. HAP1, recognized as an interactor of both HTT and SYN1, plays an essential role in replenishing the RRP, as SVs trafficking from the RP to the RRP is significantly impaired in HAP1-depleted primary cortical neurons [72, 73]. The enhanced binding of mutant HTT to HAP1 [22] may also contribute to defects in SVs mobilization from the RP to the RRP in HD.

Beyond the SVs mobilization phase, HTT is also involved in clustering SVs at the AZ through interactions with structural components of the cytomatrix at the active zone (CAZ) (Fig. 2). The CAZ scaffolding proteins Bassoon and Piccolo play a key role in positioning SVs at the AZ [74]. Co-immunoprecipitation assays in mouse cortical synaptosomes revealed that HTT associates with both Bassoon and Piccolo [75]. Notably, the formation of these complexes has been confirmed in vivo by three independent HTT interactome studies performed in the cortex and striatum of two distinct HD knock-in mice [14, 16, 17]. One of these studies demonstrates that the polyglutamine (polyQ) expansion in HTT stabilizes these interactions in the striatum during the early stages of disease progression in HD knock-in mice [16]. Another study reports that, as the disease advances, the CAZ proteins Bassoon and Piccolo are sequestered into intranuclear HTT inclusions in both transgenic mouse models and human HD cortex [76]. This sequestration disrupts the CAZ architecture and impairs synaptic vesicle mobilization from the RRP.

Recent work has expanded the protein network that regulates SVs trafficking to the AZ by including a disintegrin and metalloproteinase domain-containing protein 10 (ADAM10), a metalloprotease known for its role as the principal α-secretase in amyloid precursor protein (APP) processing and for its proteolytic regulation of synaptic cell adhesion proteins [77, 78]. While the molecular interactors of ADAM10 at the postsynapse and its synaptic substrates have been extensively elucidated [77, 79–81], its functions at the presynaptic compartment have only recently begun to emerge. Notably, interactome analyses revealed that ADAM10 binds to presynaptic proteins, including core components of the CAZ, such as Piccolo, Bassoon, ERC protein 2, and Liprin-α3 [82]. Consistently, super-resolution imaging using stimulated emission depletion (STED) microscopy demonstrated a preferential enrichment of ADAM10 at presynaptic sites, where it colocalizes with Bassoon and Piccolo and modulates SVs release at hippocampal mossy fiber boutons [83]. Interestingly, both HTT and ADAM10 are enriched on SVs in presynaptic terminals [82–84]. In vitro studies have shown that wild-type HTT binds to ADAM10 and that the formation of this complex is significantly weakened by the presence of an expanded polyQ tract in HTT, leading to increased level of active ADAM10 in HD mouse and human brains [85] (Fig. 2). Notably, an integrated analysis of HTT and ADAM10 interactome datasets revealed a striking convergence between HTT and ADAM10 interactors [82]. More than half of the proteins interacting with ADAM10 were in fact also found to interact with HTT, particularly those associated with presynaptic function [82], thereby positioning the HTT–ADAM10 complex as a key regulator of the presynaptic architecture. In the HD cortex the pathological accumulation of active ADAM10 is accompanied by a diminished binding to Piccolo [82]. Importantly, normalization of active ADAM10 level in the HD brain reinstated the ADAM10–Piccolo complex and replenished the docked pool of SVs at the AZ, indicating that the formation of this complex is functionally implicated in HD presynaptic defects [82].

Altogether, these findings delineate a dual-phase model of HTT-mediated SV recruitment at the AZ (Fig. 2). In the first phase, HTT and its adaptor HAP1 bind to SYN1 to mobilize SVs from the RP and ensure efficient RRP replenishment. In the second phase, HTT binds to ADAM10 and CAZ components—most notably Piccolo—to guide SVs from the RRP to the AZ for docking. In HD, progressive destabilization of these protein complexes impairs both phases, ultimately depleting the vesicle pools critical for effective neurotransmitter release.

#### Shaping SVs fusion

Under resting conditions, SVs fusion is tightly suppressed, but is rapidly initiated upon the arrival of an action potential. Depolarization of the presynaptic membrane during the action potential opens voltage-gated calcium channels (VGCCs), allowing calcium influx into the presynaptic terminal. Calcium ions then bind to synaptotagmin-1, a calcium sensor localized on SVs, thereby triggering the rapid and synchronous fusion of docked vesicles with the presynaptic membrane and promoting neurotransmitter release [86]. Fusion-competent SVs undergo a preparatory step known as priming, which involves the assembly of the soluble NSF attachment protein receptor (SNARE) complex, composed of three core membrane-anchored proteins: syntaxin-1 and synaptosome associated protein 25 (SNAP25) on the plasma membrane, and vesicle associated membrane protein 2 (VAMP2) on the vesicle membrane [87, 88]. This process is regulated by several accessory proteins, including Munc13-1, Munc18-1, synaptotagmin and complexin, which facilitate membrane juxtaposition and the fusion process [87, 88]. After fusion, the SNARE complexes are disassembled by a dedicated ATPase NSF, regenerating the SNARE proteins for another round of membrane fusion [87, 88].

Early studies employing co-immunoprecipitation in whole-brain homogenates of wild-type and HD transgenic mice demonstrated that HTT interacts with SNARE-associated proteins syntaxin-1A/B (STX-1A/B) and SNAP25, with no apparent difference in binding between wild-type and mutant HTT [89] (Fig. 2). Interactome analyses using immunoprecipitation combined with mass spectrometry (IP–MS) in the cortex and striatum of two HD knock-in mouse models confirmed the formation of these complexes and identified VAMP2 as an additional HTT interactor [16, 17]. Notably, one study reported a strengthened association of mutant HTT with syntaxin-1B and VAMP2 in the HD striatum [16]. An IP–MS analysis of the cortical proteome revealed that HTT also binds the SNARE proteins YKT6 and synaptosome associated protein 47 (SNAP47), and that these interactions are disrupted by an expanded polyQ tract [15]. Further evidence of impaired SVs fusion machinery in HD came from the observation that Munc13-1—a key priming factor required for SNARE complex assembly—is sequestered into mutant HTT inclusions in both mouse and human HD cortex [76]. HTT has been shown to bind also to NSF, an essential ATPase that regulates the disassembly of SNARE complex [87, 88]. This interaction was consistently identified across three independent IP-MS studies conducted in the striatum and cortex of two HD knock-in mouse models [14, 16, 17]. Moreover, mutant HTT exhibits aberrant binding to NSF in the cortex and striatum, suggesting impairments in SNARE complex disassembly and turnover in vivo [16, 17] (Fig. 2).

Palmitoylation, a post-translational modification that involves the addition of palmitic acid on cysteines, is required for the membrane localization and function of SNARE proteins, including syntaxin-1, VAMP2, and SNAP25 [90]. HTT facilitates the palmitoylation of SNARE proteins, specifically SNAP25, by interacting with Huntingtin-Interacting Protein 14 (HIP14), a neuronal palmitoyl acyltransferase (PAT) [91, 92] (Fig. 2). HIP14, identified as an HTT interactor *via* yeast two-hybrid screening, is enriched in the brain, co-localizes with HTT in the striatum, and shows reduced binding affinity in the presence of polyQ expansion [91]. Coimmunoprecipitation of HIP14 and HTT from brains of wild-type and HD transgenic mice confirmed a weaker interaction between mutant HTT and HIP14 [93]. In vitro co-immunoprecipitation assays demonstrated that the N-terminal region of HTT (residues 1–548) is necessary for its interaction with HIP14 [94]. Within this segment, two discrete binding motifs have been mapped to approximately residues 224 and 427, which are predicted to mediate the interaction with the ankyrin repeat domain of HIP14 [94]. Beyond physical interaction, HTT also modulates HIP14 enzymatic activity [95]. Disruption of the HTT–HIP14 complex results in reduced palmitoyl transferase function of HIP14 [95], leading to decreased palmitoylation of synaptic proteins such as SNAP25 in vivo, as demonstrated across four HD mouse models [96]. Ultimately, this leads to impaired SVs docking and fusion, resulting in release deficits in cortical neurons and, consequently, reduced availability of neurotrophins [56] and neurotransmitters [13, 97] to MSNs.

#### At the helm of recycling

The RRP and RP can be quickly depleted during sustained neuronal activity. The de novo biogenesis of SVs in the neuronal cell body, coupled with anterograde axonal transport to the presynaptic terminal, is typically too slow to replenish these pools in a timely manner to meet the high demand for neurotransmitter release. The local endocytosis at the presynaptic terminal becomes a critical mechanism for SVs replenishment during activity [65, 98]. Various modes of endocytosis contribute to SVs recycling. Clathrin-mediated endocytosis (CME) serves as the primary mechanism for SVs recycling under low neuronal activity conditions [98]. In contrast, during periods of heightened neuronal activity, activity-dependent bulk endocytosis (ADBE) predominates as the principal mode of SVs retrieval [65]. HTT plays a key role in modulating the complexes that govern both CME and ADBE (Fig. 2).

CME proceeds through distinct phases: nucleation, cargo selection, coat assembly, scission, and uncoating. Each step is orchestrated by key hub proteins, supported by a network of accessory factors, as extensively described over decades of research [98]. Clathrin and adaptor proteins play central roles in coat assembly and in the budding of clathrin-coated vesicles (CCVs) from the plasma membrane [98]. The first indication for a role of HTT in CME came from the identification of its interaction with HIP1 [26, 27], a protein that regulates coat assembly by binding to both clathrin adaptor protein 2 (AP2) complex and the terminal domain of clathrin heavy chain [99–102]. The AP2 complex is a heterotetramer composed of two large adaptins (α and β), a medium adaptin (μ), and a small adaptin (σ), which functions at the cell membrane to internalize cargoes in CME [98]. HTT and HIP1 are enriched in the human cerebral cortex and co-localize with clathrin heavy chain and α-adaptin on CCVs [26, 27, 99, 100]. Co-immunoprecipitation assays conducted both in vitro, using immortalized striatal cells [103], and in vivo in the mouse forebrain [104], revealed that HTT interacts with the α-adaptin subunit of the AP2 complex. Three HTT interactome studies confirm the binding between HTT and α-adaptin, and further reveal interactions with the other core components of the complex in wild-type mouse cortex and striatum [14, 16, 17]. These findings point to a functional role for HTT in coat assembly by modulating clathrin-coated pit invagination through its interactions with both HIP1 and AP2 complex. Both HTT and subunits of the AP2 complex bind to ADAM10 [85, 103, 105], suggesting that the ADAM10–HTT complex may facilitate the recruitment of AP2 to the synaptic membrane. Studies in striatal cells expressing mutant HTT, as well as in forebrain synaptosomes derived from homozygous HD knock-in mice, demonstrated a marked reduction in the interaction between mutant HTT and α-adaptin in the presence of an expanded polyQ tract [103, 104]. Moreover, yeast two-hybrid analyses revealed that the mutant form of HTT exhibits diminished binding affinity for HIP1 [26]. Although a study in a heterozygous knock-in mouse model suggests that mutant HTT binds more strongly to α- and β-adaptin than the wild-type protein in the cortex [17], these findings indicate that the pathogenic polyQ expansion in HTT disrupts clathrin coat assembly by compromising the integrity of the HTT-HIP1-AP2 complex (Fig. 2). The final step of CME, SVs scission, is catalysed by the GTPase dynamin 1 (DNM1) [65, 98, 106]. Upon recruitment to the vesicle neck, DNM1 oligomerizes into helical structures and, in concert with accessory proteins, mediates membrane fission, releasing the vesicle from the plasma membrane [65, 98, 106, 107]. Yeast two-hybrid screening revealed that DNM1 interacts with the N-terminal and C-terminal regions of HTT [89, 108]. Additional in vitro studies demonstrated that DNM1 associates with endogenous HTT, with this interaction markedly enhanced when full-length HTT contains an expanded polyQ tract [109]. DNM1 and HTT form a complex also in mouse brain, as described in an IP-MS study performed in wild-type and transgenic mice expressing full-length human mutant HTT, but how the expanded polyQ affects this interaction in vivo still need to be determined [14]. Notably, in vitro assays indicated that the C-terminal region of HTT binds to DNM1 and reduces its GTPase activity [108], thereby implicating this specific interaction in the downregulation of DNM1 enzymatic activity and SVs scission. Collectively, these results suggest that mutant HTT impairs CME by destabilizing early coat assembly and by interfering with the vesicle scission step.

ADBE is a rapid, clathrin-independent process that invaginates large regions of the plasma membrane within 1–2 s forming bulk endosomes from which SVs can bud to replenish the RP [65]. Proteomic analyses of purified bulk endosomes revealed that the Rab GTPase family plays a central role in the endosomal proteome [110]. As previously discussed, HTT forms complexes with multiple Rab GTPases in axonal trafficking [32–34, 111]; notably, other studies have extended the relevance or Rab GTPases as HTT partners (Fig. 2), particularly Rab5, Rab7, and Rab11 for their role in distinct phases of ADBE, including early endosome formation, late endosome transition, vesicle budding and recycling. Rab5 regulates early endosome biogenesis and motility [112]. Affinity chromatography assays have shown that HTT interacts specifically with the GTP-bound active form of Rab5, a binding that depends on the presence of huntingtin-associated protein 40 (HAP40) [113]. Table 2 is devoted to the HAP40–HTT complex, providing a detailed overview of its structure and functional significance. In HD models, including immortalized mutant HTT knock-in cell lines, patient-derived fibroblasts, and post-mortem brain tissues, HAP40 levels are markedly elevated [113]. This increase is accompanied by a significant accumulation of the HTT-HAP40 complex on Rab5-positive early endosomal membranes, which correlates with a reduction in endosomal motility in mutant HTT cells in vitro [113]. More recently, no abnormal accumulation of HAP40 was observed in cellular models derived from HD mice or human patients [114], highlighting the need for further investigation of the HTT–HAP40–Rab5 complex in the context of endosomal trafficking defects observed in HD. HTT and HAP40 form an obligate heteromeric complex, such that the loss of one protein leads to the destabilization and degradation of the other [114, 115]. Given their tight interdependence, the HTT-HAP40 complex may play important, yet unexplored, roles at the synapse, possibly extending beyond endosomal trafficking. During endosome maturation, Rab5 is gradually replaced by Rab7, which is recruited to late endosomal membranes [112]. Rab7 regulates the transition from early to late endosomes and their subsequent fusion with lysosomes [112]. In neurons, Rab7 is particularly important in balancing SVs recycling with protein degradation, which is critical for maintaining synaptic integrity and homeostasis [112]. Studies in *Drosophila melanogaster* larval axons have shown that HTT interacts with Rab7 and lysosomal associated membrane protein 1 (LAMP1) on late endosomes [33]. Co-immunoprecipitation analyses, performed on the mouse brain, have confirmed the existence of the HTT–Rab7–LAMP1 complex in mammalian systems [33], positioning HTT in ADBE as a modulator of the endo-lysosomal pathway in coordination with Rab7. Rab11 is another critical partner of HTT in ADBE. Rab11 coordinates the budding of recycling endosomes from early endosomes and their trafficking back to the plasma membrane, ensuring efficient recycling of membrane proteins [112]. Biochemical studies have shown that HTT co-precipitates with the GDP-bound inactive form of Rab11 in mouse brain membrane fractions [116]. Remarkably, experiments in HTT-null embryonic stem cells revealed that HTT is essential for activating Rab11, facilitating the conversion of Rab11-GDP to its active GTP-bound form [116]. In HD, Rab11 activity is significantly reduced in whole-brain and striatal tissue from HD knock-in mice, suggesting that the expanded polyQ in mutant HTT impairs Rab11 activation and its endosomal association [117]. In HD fibroblasts, defective SVs budding from endosomes [118] could reflect a disruption of ADBE dynamics. Additionally, reduced Rab11 activity correlates with impaired recycling of the glutamate/cysteine transporter EAAC1 in cortical neurons from HD knock-in mice [119]. Since EAAC1 mediates cysteine uptake, a rate-limiting step in glutathione biosynthesis, its reduced membrane level results in intracellular glutathione depletion, compromised antioxidant capacity, and increased reactive oxygen species (ROS) in HD neurons [119]. Restoring Rab11 activity rescues EAAC1 recycling and glutathione levels, enhances ROS clearance, and improves HD cortical neuron survival [119]. Interestingly, overexpression of Rab11 reversed the synaptic transmission and vesicle trafficking deficits observed in a *Drosophila melanogaster* model of HD [120], further linking this protein to synaptic dysfunction in HD.

**Table 2:** The HTT-HAP40 complex: unlocking the secrets of a duo.

Discovery and abundance	
HAP40 was identified through its ability to co-immunoprecipitate with full-length HTT from rat brain extracts.	[196]
HAP40 is established as the most abundant HTT interactor in the mouse brain.	[14, 15]
**Structure and evolution**	
Structure of the HTT-HAP40 complex is resolved at high resolution by cryo-EM.	[197]
Strong co-evolution between HTT and HAP40 supports the functional importance of the HTT-HAP40 complex.	[198]
HAP40 stabilizes HTT which adopts a compact, globular conformation with three domains: N-terminal HEAT-repeat (N-HEAT), central bridge domain and C-terminal HEAT-repeat (C-HEAT).	[114, 115, 197, 199]
HAP40 is enclosed between the N- and C-HEAT domains of HTT and binds preferentially to its C-terminal region, with ten evolutionary conserved intermolecular contacts that stabilize the complex.	[200]
HTT and HAP40 form an obligate heteromeric complex. Loss of HTT causes rapid proteasomal degradation of HAP40, while loss of HAP40 determines reduced HTT stability.	[114, 115]
**Role in Huntington’s disease**	
Structural studies indicate minimal effects of the expanded polyQ tract on HTT–HAP40 architecture and stability.	[199, 201]
Co-immunoprecipitation studies revealed increased binding of HAP40 to mutant HTT in the brains of HD knock-in mice.	[202]
HTT-HAP40 complex is implicated in protein degradation via the ubiquitin-proteasome system.	[115, 202]
HAP40 depletion in HD mice exacerbates mutant HTT aggregation and neuronal loss, whereas HAP40 overexpression reduces aggregation and ameliorates behavioural deficits.	[202]

#### Regulating the synaptic cleanup

Presynaptic proteins, primarily synthesized in the neuronal soma and transported to synaptic terminals, have relatively short half-lives and require continuous, tightly regulated turnover to sustain synaptic function [121]. Hence, damaged or misfolded presynaptic proteins must be efficiently removed via the autophagic pathway and replaced by newly synthesized proteins in a spatially and temporally orchestrated manner [122]. HTT has a role in selective autophagy, a process distinguished from bulk autophagy by its reliance on cargo specificity. Selective autophagy necessitates a cohort of receptor proteins, which identify and bind ubiquitinated substrates, facilitating their sequestration into the autophagic machinery [123]. Among these receptors, p62 occupies a central position by bridging ubiquitinated cargo with LC3 proteins located on the nascent autophagosomal membrane, known as the phagophore [123]. Autophagosome formation is initiated at the phagophore through activation of the unc-51 like autophagy activating kinase 1 (ULK1) complex, whose activity is negatively regulated by mammalian target of rapamycin complex 1 (mTORC1) [123]. Evidence from in vitro studies indicates that wild-type HTT co-immunoprecipitates with both p62 and ULK1, suggesting the formation of a ternary HTT–p62–ULK1 complex [124, 125] (Fig. 2). HTT also interacts with LC3 via its predicted LC3-interacting region (LIR) motifs [124], thus further facilitating binding of the target cargo to phagophores. More recently, a putative ubiquitin-binding domain (residues 235–367) has been identified within HTT, suggesting a direct role of this protein in cargo recruitment [126]. Through its interaction with p62, LC3 and ubiquitin, HTT promotes the recognition of ubiquitinated cargo. In addition, binding of HTT to ULK1 disrupts its interaction with mTORC1, thereby facilitating the tethering of target cargo to the autophagic pathway [124, 125]. In HD, mutant HTT disrupts these finely tuned interactions, leading to autophagy dysfunction. In vitro studies have shown that mutant N-terminal HTT fragments exhibit increased affinity for ubiquitin, which may result in the sequestration of ubiquitinated proteins into insoluble aggregates, rendering them inaccessible to p62 and thereby impeding selective autophagy [126]. Moreover, the interaction between mutant HTT and p62 appears to be dysregulated. While one study in HD knock-in cells reported increased binding between mutant HTT and p62 [127], another study using overexpression systems observed reduced binding [128]. These discrepancies may reflect differences in experimental models and protein expression levels, but in either case, the altered interaction between mutant HTT and p62 likely compromises p62-mediated cargo recognition and delivery to the autophagosome. Mutant HTT has been shown to bind less efficiently to ULK1 in HD knock-in cells, resulting in sustained ULK1 inhibition via mTORC1 and reduced autophagy induction [129]. Autophagic dysfunction is further exacerbated by defects in the retrograde transport of autophagosomes, which fail to reach the soma for efficient lysosomal degradation [41]. Furthermore, studies in HD knock-in cell lines have shown that the interaction between mutant HTT and two essential mitophagy receptors - optineurin and calcium binding and coiled-coil domain-containing protein 2 (CALCOCO2) - is reduced, thereby impairing the clearance of damaged mitochondria [129], a defect that may have significant consequences also at the synaptic level.

## Mapping postsynaptic hips

Beyond its multifaceted role in presynaptic regulation, HTT is also essential for maintaining optimal postsynaptic architecture and function, primarily through its interactions with a diverse array of postsynaptic proteins. In the following sections, we examine HIPs involved in the assembly of postsynaptic scaffolds, receptor trafficking and recycling, and the regulation of synaptic cell adhesion.

### HIPs in intracellular receptor transport and recycling

**Fig. 3:**
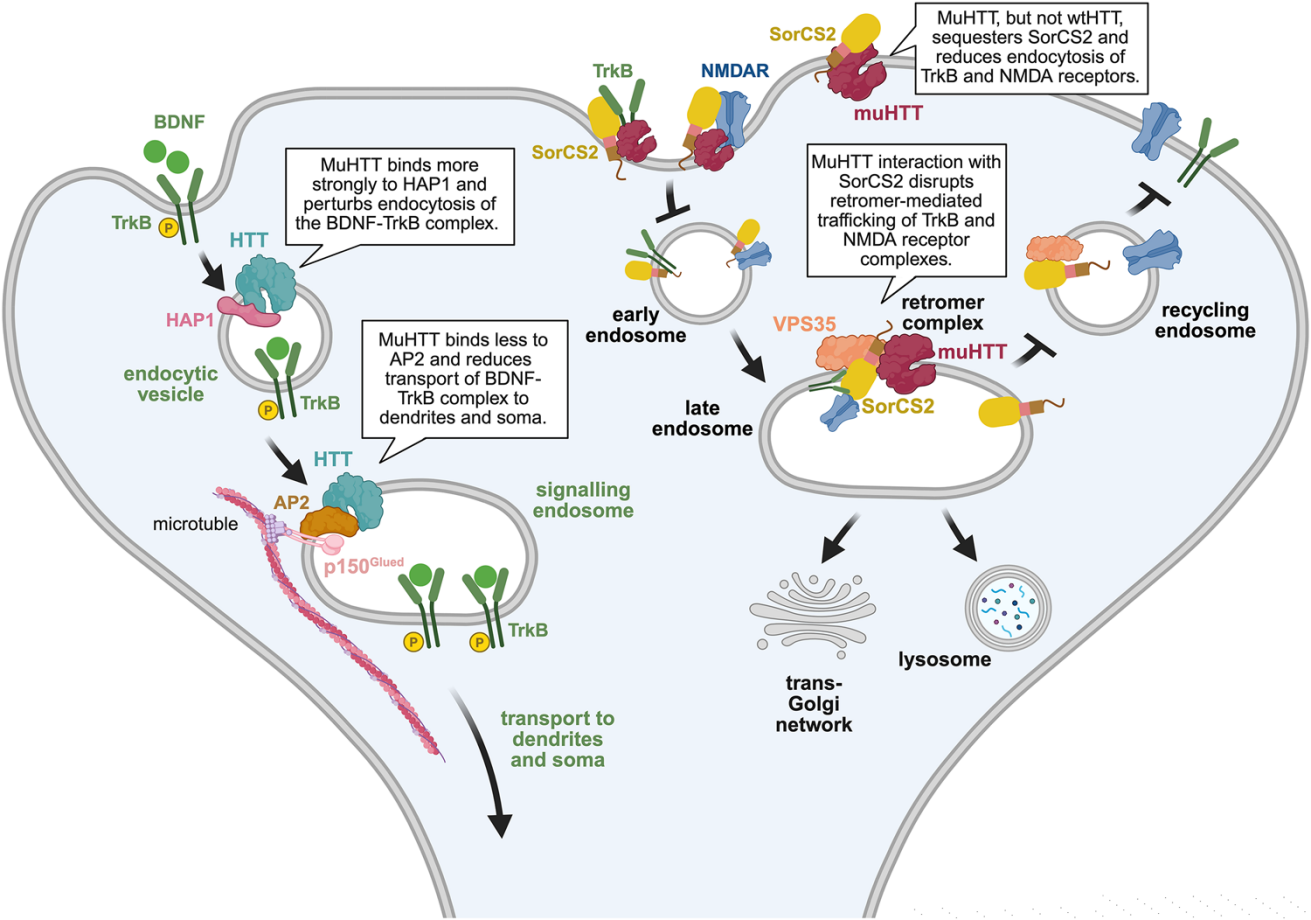
HTT interacting proteins in signalling endosome transport and receptor recycling. HIPs and signalling endosomes: following BDNF binding, phosphorylated TrkB (P-TrkB) is internalized into endosomes by the HTT-HAP1 complex. HTT binding to the AP2-p150^Glued^ complex facilitates retrograde transport of signalling endosomes. The formation of these complexes is disrupted in the presence of mutant HTT (muHTT), leading to reduced endosome transport. HIPs and receptor recycling: by recruiting SorCS2 mutant HTT reduces TrkB and NMDARs endocytosis. Additionally, mutant HTT binding to SorCS2 within the retromer complex impairs receptor recycling. *Created in* https://BioRender.com/r55y183.

HTT plays a pivotal role in regulating the abundance of surface receptors at the postsynaptic membrane (Fig. 3). In striatal MSNs, wild-type HTT – either directly, via kinesin-1 heavy chain KIF5 and dynein, or through its adaptor HAP1 – coordinates the bidirectional movement of key receptor cargoes along microtubules [31, 43, 130, 131]. In contrast, mutant HTT destabilizes these trafficking complexes, culminating in a sharp reduction of receptor density at the postsynaptic surface [31, 43, 130, 131]. Notably, this includes impaired trafficking of α-amino-3-hydroxy-5-methyl-4-isoxazolepropionic acid (AMPA), gamma-aminobutyric acid type A (GABA-A), TrkB, and TrkA receptors, which are core mediators of excitatory transmission, inhibitory balance, and neurotrophic support [31, 43, 130, 131], although it is likely that additional receptor systems will emerge as being affected in future studies. These deficits compromise synaptic responsiveness and plasticity, leaving MSNs increasingly vulnerable to dysfunction and degeneration.

HTT involvement in maintaining proper receptors abundance at the postsynapse is further documented by its binding to sortilin-related VPS10 domain-containing receptor 2 (SorCS2), whose function is to bind receptors and promote their endocytosis and recycling [132]. SorCS2 is selectively expressed in the MSNs of the dorsal striatum and has a strong affinity for TrkB and the GluN2B subunit of N-methyl-D-aspartate receptors (NMDARs) [133–136]. In the mouse HD striatum, mutant HTT sequesters SorCS2, impairing its function during the early stages of endosomal recycling, resulting in a reduction of early endosomes carrying TrkB and NMDA receptors [132, 136] (Fig. 3). Endosomal maturation and receptor recycling depend on recruitment of the retromer complex, a key trafficking machinery whose central component, VPS35, interacts with SorCS2 to direct receptor cargoes back to the postsynaptic membrane [137]. In addition to the overall reduction of SorCS2 protein levels, a selective aberrant interaction between mutant HTT and SorCS2 has been described in the striatum of HD knock-in mice [132]. As a result of this interaction, the retromer complex fails to recruit the TrkB-SorCS2 and NMDAR-SorCS2 complexes in HD, resulting in significantly decreased recycling of these receptors to the dendritic surface of MSNs [132, 136] (Fig. 3). While it is well-established that genetic deficiency of SorCS2 accelerates the onset and exacerbates behavioural deficits in two mouse models of HD [132, 136], the therapeutic potential of targeting SorCS2 or other components of the retromer complex remains to be fully explored. Retromer-stabilizing compounds have already proven effective in mitigating synaptic plasticity loss and cognitive decline in mouse models of Parkinson’s and Alzheimer’s diseases [138–140]. Stabilization of the retromer complex may also represent a potential avenue for investigation in HD.

In parallel, HTT is also implicated in the regulation of signalling endosomes, specialized organelles that sustain intracellular neurotrophic signalling [141], as exemplified by the BDNF–TrkB complex. Upon binding with cortical-derived BDNF, phosphorylated TrkB on MSNs undergoes endocytosis into endosomes, where it continues signalling within the cell soma, dendrites, and axons [141–143]. This process ensures synapse maintenance, dendritic arborization, axonal growth, and survival of striatal neurons [141–143]. Evidence for HTT participation in the regulation of signalling endosomes in striatal neurons has emerged, emphasizing specific protein partners involved in this process (Fig. 3). In particular, by binding to HAP1 and the AP2-p150^Glued^ complex, HTT enables endocytosis and retrograde transport of active BDNF-TrkB complex from the postsynaptic membrane to the soma in primary striatal neurons [43, 143]. Interestingly, the conventional roles of HAP1 in trafficking and AP2-p150^Glued^ in endocytosis appear reversed in these complexes: HTT–HAP1 is essential for endocytosis, whereas HTT–AP2– p150^Glued^ mediates the transport of BDNF–TrkB-containing endosomes [43, 142, 143]. In HD, mutant HTT displays heightened affinity for HAP1 [24] and reduced interaction with core components of the AP2 complex has been found in the striatum [103], thereby disrupting both endocytosis and retrograde transport of the active BDNF-TrkB complex to the MSNs soma. These intracellular trafficking defects, together with the reduced pool of cortical-derived BDNF, further impair BDNF signalling in MSNs, thereby increasing striatal vulnerability.

### HIPs guiding NMDARs to synaptic and extrasynaptic sites

**Fig. 4:**
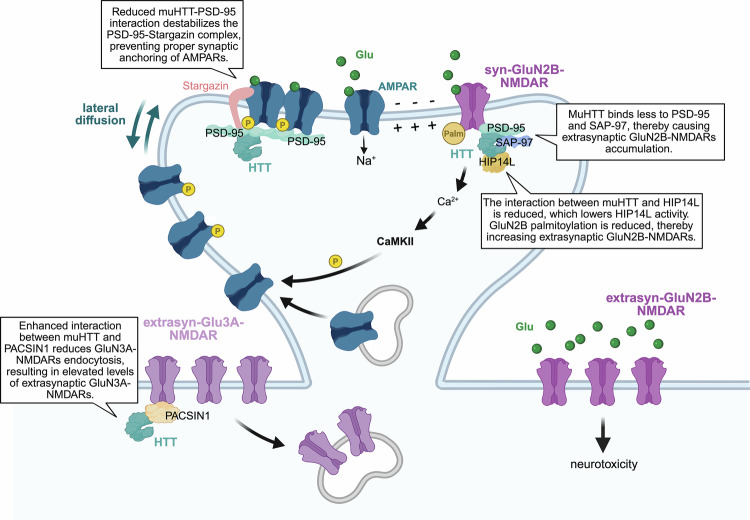
HTT partners regulating NMDARs localization and AMPARs lateral diffusion. HTT regulates the synaptic localization of GluN2B-containing NMDARs through interactions with PSD-95 and SAP-97. Mutant HTT (muHTT) shows reduced binding to these scaffolds, leading to accumulation of extrasynaptic GluN2B-NMDARs and neurotoxicity. HTT also associates with HIP14L, which palmitoylates GluN2B to stabilize synaptic GluN2B-NMDARs. This interaction is disrupted by mutant HTT, leading to decreased GluN2B palmitoylation and enhanced localization of GluN2B-NMDARs at extrasynpatic sites. Additionally, HTT binds to PACSIN1 to regulate extrasynaptic Glu3A-NMDARs endocytosis. Mutant HTT binding to PACSIN1 is increased, resulting in reduced endocytosis of GluN3A-containing NMDARs at extrasynaptic sites. HTT also regulates AMPARs dynamics by interacting with the PSD-95–Stargazin complex to stabilize AMPARs at synapses. In HD, disruption of this complex impairs LTP. *Created in* https://BioRender.com/r55y183.

Over the last two decades, extensive research has demonstrated that HTT is implicated in the localization of NMDARs between synaptic and extrasynaptic sites, with profound consequences on synaptic plasticity and neuronal survival [144–146] (Fig. 4). The functional outcome of NMDARs activation is highly dependent on their subcellular localization. The stimulation of synaptic NMDARs, which act primarily through nuclear Ca²⁺ signalling, results in a neuroprotective “shield” [144]. Conversely, the stimulation of extrasynaptic NMDARs promotes excitotoxicity [144]. This dichotomy arises from the engagement of distinct transcriptional programs with opposing effects on intracellular signalling cascades [144]. In HD, striatal neurons exhibit a marked shift in the ratio of extrasynaptic to synaptic GluN2B-containing NMDARs, thereby enhancing the vulnerability of these neurons to excitotoxic insults [145, 146]. Key partners of HTT in this process are the postsynaptic density protein 95 (PSD-95), synapse-associated protein 97 (SAP-97), and the palmitoyltransferase huntingtin-interacting protein 14-like (HIP14L), a paralog of HIP14 with identical domain structure (Fig. 4).

PSD-95 organizes glutamatergic synapses by anchoring NMDARs and AMPARs through its three PDZ domains [147]. Co-immunoprecipitation experiments performed in vitro in cells overexpressing PSD-95 revealed for the first time the formation of a complex between HTT and this scaffolding protein [148]. The same study showed that the proline-rich domain (PRD) of HTT binds directly to the SH3 domain of PSD-95 in vitro [148]. However, the polyQ expansion in mutant HTT disrupts this interaction, leading to reduced HTT-PSD-95 complex formation in the striatum of HD mice and in the human HD cortex [148, 149]. Consequently, PSD-95 accumulates at extrasynaptic sites, where it stabilizes GluN2B-NMDARs and amplifies excitotoxic signalling cascades, contributing to synaptic degeneration and MSNs loss [145, 146, 150, 151].

It is worth noting that the postsynaptic scaffolding proteins PSD-95 and SAP-97 have recently emerged from two HTT interactome studies as HTT partners together with other scaffolding proteins [16, 17]. Notably, overexpression of SAP-97 prevents the pathological shift of GluN2B-NMDARs to extrasynaptic sites in HD hippocampal neurons and reduces neuronal death [152]. These findings suggest a model in which HTT becomes a structural component of the PSD compartment, acting as a hub to coordinate the recruitment of scaffolding molecules such as PSD-95 and SAP-97. Reduced mutant HTT binding to PSD-95 [148], and possibly to other scaffolding proteins such as SAP-97, can have a profound effect on receptors clustering and location in HD.

HTT regulates synaptic NMDARs localization also through its influence on protein palmitoylation. This process is mediated by the palmitoyl acyltransferases HIP14 and HIP14L, which are responsible for the palmitoylation and synaptic stabilization of GluN2B-containing NMDARs [153]. The yeast two-hybrid system and co-immunoprecipitation studies in mammalian cells revealed that wild-type HTT directly interacts with both HIP14 and HIP14L, and this interaction is essential for their full enzymatic activity [91, 94, 95]. It was also shown that the HTT N-terminal region encompassing amino acids 1–548 is sufficient for full interaction with both HIP14 and HIP14L [94]. Two potential binding sites for HIP14 have been identified within this HTT region, localized around residues 224 and 427 [94]. The HTT–HIP14 complex appears to function as a regulatory unit that ensures proper palmitoylation of GluN2B subunits, thereby promoting their retention at synaptic sites. Mutant HTT shows reduced affinity for HIP14L, leading to a loss of enzymatic function and impaired GluN2B palmitoylation [91, 94, 150, 154]. This defect results in the mislocalization of GluN2B-NMDARs to extrasynaptic sites, causing excitotoxic signalling and neuronal vulnerability in the striatum [150, 154] (Fig. 4).

In addition to GluN2B-NMDARs, GluN3A-containing NMDARs (GluN3A-NMDARs), a developmentally regulated and less-characterized receptor subtype, abnormally accumulate at extrasynaptic sites in the HD brains [155]. Expression of GluN3A-NMDARs is highest during early postnatal and juvenile stages, decreasing in adulthood [156]. The endocytic removal of GluN3A-NMDARs from the postsynaptic membrane is mediated by protein kinase C and casein kinase substrate in neurons 1 (PACSIN1), an endocytic adaptor that binds directly to the GluN3A C-terminus [155, 157] (Fig. 4). Although PACSIN1 interacted with both HTT variants in striatal lysates from wild-type and HD transgenic mice, the interaction was stronger in the HD striatum [155]. In vitro studies indicated that the PRD of HTT interacts with the C-terminal SH3-domain of PACSIN1, and that this interaction is enhanced by the polyQ expansion present in mutant HTT [155, 157]. This increased binding leads to the sequestration of PACSIN1 by mutant HTT, which in turn blocks the endocytosis of GluN3A-NMDARs [155, 157]. As a result, there is an unexpected and neurotoxic accumulation of GluN3A-NMDARs at extrasynaptic sites in HD [155]. Notably, genetic deletion of GluN3A in HD transgenic mice has been shown to restore synaptic integrity and reverse both motor and cognitive deficits [155], underscoring the functional importance of GluN3A-NMDARs mislocalization in HD pathogenesis. However, recent findings also point to a potentially protective, compensatory role for GluN3A-NMDARs under conditions of acute stress [156]. These observations suggest that while extrasynaptic GluN3A-NMDARs activation may contribute to HD pathology, its precise role remains complex and warrants further investigation.

### HIPs anchoring AMPARs

Elegant experiments using primary hippocampal neurons from HD mouse models have demonstrated a role for the HTT–PSD95 complex in regulating AMPARs lateral diffusion during long-term potentiation (LTP) [158]. LTP is triggered by the activation of postsynaptic NMDARs and is expressed through an increase in the synaptic abundance of AMPARs complexes [159, 160]. Following LTP induction, phosphorylated AMPARs accumulate at extrasynaptic sites and diffuse laterally to synaptic sites to be trapped and stabilized at the PSD [159, 160]. The diffusional trapping of surface AMPARs is crucial to ensure the rapid potentiation of synaptic transmission during LTP expression [159, 160]. In HD, mutant HTT leads to increased AMPARs surface diffusion but AMPARs fail to stabilize at the PSD after LTP induction [158]. The underlying mechanisms involve the destabilization of the PSD-95–Stargazin complex [158] (Fig. 4). Stargazin is one of four homologous transmembrane AMPARs regulatory proteins (TARPs), fundamental for AMPARs membrane anchoring [159, 160]. HTT, through its interaction with PSD-95, is also part of this protein complex and contributes to regulate AMPARs anchoring at the PSD [148, 149]. In HD, reduced binding of mutant HTT to PSD-95 [148, 149] may disrupt the PSD-95–Stargazin complex, thereby compromising AMPARs anchoring and stabilization at the postsynaptic membrane.

Interestingly, cortical BDNF signalling also emerges as a crucial upstream modulator of postsynaptic scaffold stability and receptor dynamics in HD. BDNF released from cortical afferents stabilizes PSD-95 at the synapse [161]. In HD models, the well-documented reduction in cortical BDNF contributes to disassembly of the PSD-95–Stargazin complex in hippocampal neurons, leading to abnormal surface diffusion of AMPARs during LTP [158]. Notably, pharmacological enhancement of BDNF signalling, via the antidepressant tianeptine, restores the formation of the PSD-95–Stargazin complex and slows down the increased AMPARs surface diffusion in HD neurons [158]. These findings highlight the importance of presynaptic-derived BDNF not only in synaptic communication but also in maintaining the structural and functional integrity of the postsynaptic architecture.

### HTT-ADAM10 interplay in synaptic cell adhesion

**Fig. 5:**
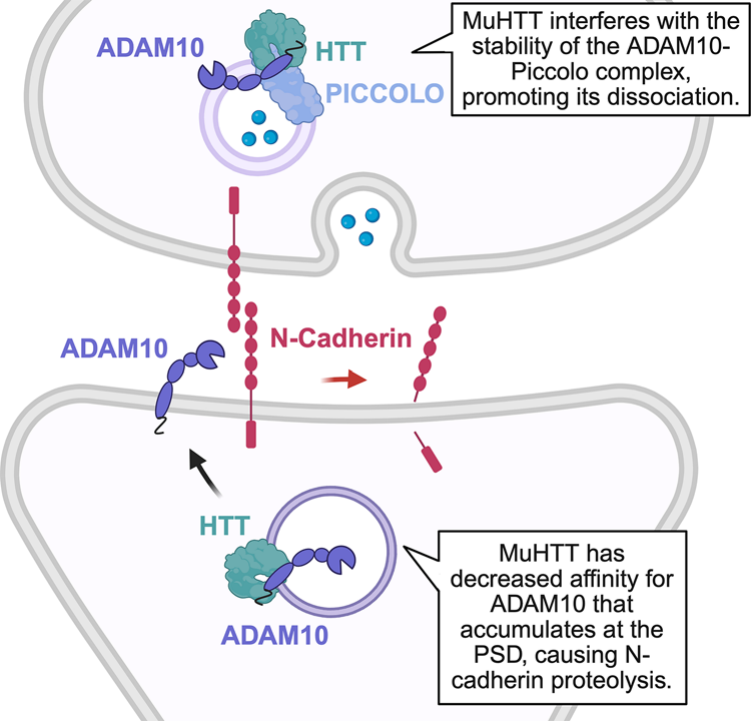
The dual role of the HTT-ADAM10 complex at the synapse. At the postsynaptic site, HTT interacts with mature, catalytically active ADAM10 to regulate its proteolytic activity toward synaptic adhesion proteins, such as N-cadherin. In HD, mutant HTT (muHTT) shows reduced affinity for active ADAM10, leading to its accumulation at the postsynaptic density (PSD) and excessive cleavage of N-Cadherin, which weakens synaptic adhesion. At the presynaptic site, HTT and ADAM10 share interactors involved in synaptic vesicle (SV) homeostasis, including Piccolo. Elevated level of active ADAM10 in HD disrupts the ADAM10–Piccolo complex, thereby reducing the pool of SVs at presynaptic terminals. *Created* https://BioRender.com/r55y183.

Beyond their classical role as architectural scaffolds during synaptogenesis, synaptic adhesion molecules have emerged as dynamic regulators of excitatory neurotransmission and synaptic plasticity in the mature brain, mediating their effects through intimate molecular dialogues with AMPA and NMDA receptors [162, 163]. Central to this regulatory axis is the metalloprotease ADAM10 that, by controlling the proteolytic processing of adhesion molecules at the postsynaptic membrane, is recognized as a critical determinant of synaptic plasticity [77, 164–166]. Recent insights, mostly derived from the work of some of the authors, have expanded this paradigm, implicating HTT as a modulator of synaptic adhesion dynamics *via* its association with ADAM10 [85, 167]. Co-immunoprecipitation experiments in mammalian cells indicated that wild-type HTT forms a complex with ADAM10, regulating its proteolytic activity on synaptic cell adhesion protein N-cadherin [85, 167]. However, in the presence of a pathogenic polyQ expansion, the formation of HTT-ADAM10 complex is reduced, and the level of active ADAM10 increases, culminating in abnormal proteolytic cleavage of N-cadherin [85] (Fig. 5). Increased N-cadherin proteolysis has been observed in the brains of two HD mouse models and in the HD human caudate, and is closely associated with deficits in excitatory synaptic transmission [85]. N-cadherin, through its extracellular domain, engages in heterophilic interactions with AMPARs, regulating their lateral diffusion and facilitating dendritic spine formation [168]. LTP induction selectively promotes the trapping of GluA1-containing AMPARs at the spine head, a process fundamental for synaptic strengthening [169, 170]. In HD hippocampal neurons, the pathological accumulation of active ADAM10 and the consequent loss of N-cadherin integrity induced a dramatic depletion of mushroom spines containing GluA1-AMPARs [171]. Strikingly, ADAM10 inhibition increased the density of mushroom spines enriched with GluA1-AMPARs and prevented LTP defects in HD hippocampal neurons [171]. These findings, together with prior evidence identifying the HTT-ADAM10 complex as a regulator of presynaptic function [82, 83], indicate that its disruption in HD perturbs the homeostatic balance of the pre- and postsynaptic compartments (Fig. 5). This dual role of ADAM10 at the synapse highlights its importance as a HIP and a potential target for mitigating synaptic dysfunction in HD.

## Htt–lipid crosstalk with a focus on cholesterol in synaptic function

Lipids are fundamental to synaptic architecture and function, with HTT positioned as a central regulator of their homeostasis. By binding membrane phospholipids and modulating cholesterol biosynthesis, one of the most abundant and indispensable synaptic lipids, HTT directly couples lipid metabolism to synaptic integrity.

At molecular level, HTT interacts with phospholipids via its amphipathic N-terminal 17 (N17) domain and a secondary lipid-binding region between residues 172–372 [ref. 172], enabling its association with a variety of lipid membranes ranging from the plasma membrane to specific organelles and SVs [173, 174]. Mutant HTT exhibits increased affinity for phospholipids and forms pore-like assemblies that disrupt membrane integrity [175, 176]. This damage can extend to vesicular and synaptic membranes, impairing synaptic structure and function. HTT–lipid interactions are also highly sensitive to membrane composition: cholesterol, which regulates membrane curvature [177], fluidity [178] and thickness [179] can either promote or inhibit mutant HTT binding to membranes, depending on the local lipid environment [180].

In the CNS, where the blood–brain barrier prevents peripheral cholesterol uptake, astrocytes provide the main source of de novo cholesterol synthesis, tightly coupling neuronal metabolism to astrocytic cholesterol supply [181]. This biosynthetic pathway is regulated at the transcriptional level by sterol regulatory element-binding protein 2 (SREBP2), a master transcription factor governing cholesterol metabolism [182]. Cholesterol biosynthesis genes typically contain sterol regulatory elements (SREs) within their promoters, allowing direct transcriptional regulation by SREBP2 [182]. In mammalian cells mutant HTT interacts with the SREBP2–Importin-β complex and impairs its translocation into the nucleus [183]. In addition, other studies showed that the active N-terminal form of SREBP2 is present at reduced levels in the nuclear fractions of cells and mouse brains expressing mutant HTT, leading to attenuated expression of cholesterol biosynthesis genes and, consequently, diminished cholesterol levels [181].

Cholesterol is a structural component of the AZ and is indispensable for the SV cycle, regulating SVs biogenesis, docking, release and recycling [184, 185]. It organizes lipid rafts, cholesterol- and sphingolipid-rich microdomains that serve as hubs for receptor localization and signalling [185]. Cholesterol also binds to a cholesterol recognition/interaction amino acid consensus (CARC) motif present in TrkB, the high-affinity receptor for BDNF, thereby modulating its activation [186]. This mechanism is particularly relevant at cortico-striatal synapses, where BDNF–TrkB signalling plays a central role in maintaining synaptic strength and plasticity [52–55]. Notably, a wealth of evidence demonstrates cholesterol reduction in the mouse HD brain, and exogenous cholesterol administration has been shown to exert neuroprotective effects across multiple pathological parameters in HD, to the extent of supporting its potential therapeutic application [187].

Beyond cholesterol, levels of the ganglioside GM1 – which promotes spine formation and synaptic plasticity [188]—are also markedly reduced in the brains of HD mice [189]. Notably, GM1 administration provides neuroprotection in HD models [190, 191]. Together, the depletion of both cholesterol and GM1 in HD may synergistically destabilize HTT–lipid interactions, thereby driving profound remodelling of synaptic membranes, SVs cycling, receptor dynamics, and intracellular signalling within the cortico-striatal circuitry.

## Concluding remarks and future perspectives

This review summarizes the extensive repertoire of pre- and postsynaptic protein interactors of HTT. Among these, presynaptic HIPs, strategically positioned within vulnerable cortical neurons – recognized as primary drivers of striatal degeneration in HD – emerge as particularly compelling targets. Despite this premise, several critical gaps remain. Expanding the validation of presynaptic HIPs specifically within human post-mortem cortical tissue and in HD cortical neurons derived from human pluripotent stem cells is a crucial step to bridge mechanistic insights with translational relevance. Similarly, clarifying the role of HTT PTMs in modulating HTT binding to presynaptic HIPs may provide important cues. It would also be valuable to explore whether alterations in HTT–lipid interactions influence the composition or dynamics of the HTT synaptic interactome. Nanoscale imaging technologies offer unprecedented opportunities to visualize proteins in situ, including at single-molecule resolution [192, 193], thereby revealing critical structural insights of HTT and its partners within pre- and postsynaptic compartments. In parallel, microfluidic platforms, enabling compartmentalized fluidic isolation and independent analysis of circuitry components [194], represent a powerful strategy to dissect the functional contributions of cortical presynaptic HIPs to HD cortico-striatal dysfunction. Collectively, a focused investigation on presynaptic HIPs has the potential to substantially deepen our mechanistic understanding of HD pathogenesis and, importantly, to foster the development of novel, synapse-targeted therapeutic strategies tailored to cortical neurons.
